# Effectiveness of Intravenous Non-Opioid Analgesics for Postoperative Pain Management of in Patients Undergoing Hip Surgery: A Systematic Review and Meta-Analysis

**DOI:** 10.3390/medicina59111904

**Published:** 2023-10-27

**Authors:** Jae Suk Chang, Dong Hwan Lee, Min Wook Kang, Ji Wan Kim, Chul-Ho Kim

**Affiliations:** 1 Department of Orthopedic Surgery, National Police Hospital, Seoul 05715, Republic of Korea; jschang3525@gmail.com (J.S.C.); gopress@hanmail.net (D.H.L.); npng4eve@gmail.com (M.W.K.); 2 Department of Orthopedic Surgery, Asan Medical Center, University of Ulsan College of Medicine, Seoul 05505, Republic of Korea; bakpaker@hanmail.net

**Keywords:** intravenous, non-opioid analgesics, acetaminophen, NSAIDs, hip surgery

## Abstract

*Background and Objectives*: Intravenous (IV) non-opioid analgesics (NOAs) have been extensively investigated as a multimodal analgesic strategy for the management of acute pain after hip surgery. This pair-wise meta-analysis examined IV NOA effects following hip surgery. *Materials and Methods*: A systematic search of the MEDLINE (PUBMED), Embase, and Cochrane Library databases was performed for studies investigating the effect of IV NOA for postoperative pain management following hip surgery up to 7 June 2023. We compared in-admission opioid use, postoperative VAS (visual analogue scale) score, hospital stay duration, and opioid-related adverse events between IV NOA and control groups. *Results*: Seven studies were included with a total of 953 patients who underwent hip surgery. Of these, 478 underwent IV NOA treatment, and 475 did not. The IV NOA groups had lower opioid use within 24-h following hip surgery (SMD, −0.48; 95% CI, −0.66 to −0.30; *p* < 0.01), lower VAS score (SMD, −0.47; 95% CI, −0.79 to −0.16; *p* < 0.01), shorter hospital stay (SMD, −0.28; 95% CI, −0.44 to −0.12; *p* < 0.01), and lower incidence of nausea and vomiting (OR, 0.32; 95% CI, 0.15 to 0.67; *p* < 0.01) compared with the control groups. *Conclusions:* This meta-analysis demonstrated that IV NOA administration following hip surgery may have more favorable postoperative outcomes than those in control groups.

## 1. Introduction

As the elderly population continues to grow, the global frequency of hip surgery is progressively increasing [1]. According to prior research, over 1.5 million patients worldwide are diagnosed with hip fractures annually [2].

Effective postoperative pain control after hip joint surgery reduces the occurrence rate of complications after surgery and is associated with functional recovery after surgery [3,4]. Elderly patients undergoing hip surgery have a higher risk of developing postoperative delirium, chronic pain, ambulation difficulties, and in-hospital fatalities, particularly with ineffective postoperative pain management [5]. Therefore, effective analgesia is critical for patients undergoing hip surgery.

Historically, opioids have been widely used for perioperative pain management, not only in hip joint surgery but also in orthopedic surgery more generally [6]. A study based on 2014–2017 United States (U.S.) population data reported an increase in the rate of opioid prescriptions from 82.0% to 89.7% within 60 days after total hip arthroplasty [7]. However, the potential overprescribing of opioids after surgery may contribute to serious opioid-related adverse events [8,9]. These include nausea, vomiting, urinary retention, and constipation, as well as more severe effects such as deep sedation and, in extreme cases, respiratory depression [4,10]. Accordingly, since 2014, various institutions in the U.S., including the U.S. Drug Enforcement Administration, the U.S. Centers for Disease Control, and state governments, have implemented measures to restrict opioid overprescribing [11,12]. Multimodal analgesia strategies are gaining increasing attention as an additional method to reduce opioid use.

Intravenous (IV) non-opioid analgesics (NOAs) have been extensively investigated as a multimodal analgesic strategy for managing acute pain following orthopedic surgery [4]. These include IV non-steroidal anti-inflammatory drugs (NSAIDs) and IV acetaminophen.

Numerous studies have examined the effectiveness of IV NOAs, but there are limited large-scale studies or randomized controlled trials (RCTs) in the field of orthopedic surgery and, specifically, hip surgery. This study conducted a pair-wise meta-analysis on the effects of IV NOAs following hip surgery.

The hypothesis of this study was that acute and overall opioid use is lower in patients undergoing IV NOA treatment than in patients not treated with IV NOAs. Additionally, we postulated that the VAS (visual analogue scale) score, postoperative hospital stay duration, and opioid-related side effects would also be lower in the group undergoing IV NOA treatment.

## 2. Materials and Methods

This systematic review and meta-analysis followed the preferred reporting items for systematic reviews and meta-analyses guidelines. [13,14] While this analysis involved human participants, both ethical approval and the acquisition of informed consent from participants were not required because all data were based on previously published studies and were anonymously analyzed without any harm to the participants.

### 2.1. Literature Search

In compliance with the referenced guidelines, we searched MEDLINE, Embase, and the Cochrane Library for studies investigating the outcomes of IV NOA use for postoperative pain management of hip surgery. Using an a priori search strategy, we identified articles published up to 7 June 2023. Search terms included synonyms and terms related to hip surgery and IV NOAs. The full search strategies and results for all databases are presented in Appendix A. We placed no restrictions on language or publication year. After the initial electronic search, we manually searched the relevant articles and associated bibliographies.

### 2.2. Study Selection

Two board-certified orthopedic surgeons who worked as faculty members at an academic medical center independently selected the articles for full-text review from the titles and abstracts of the studies. If the abstract provided insufficient data to finalize a decision, the entire article was reviewed.

This meta-analysis was designed as a pairwise meta-analysis. Studies were included based on the “Populations/Intervention/Comparator/Outcome/Study design” (PICOS) criteria [15]: (1) “populations” were set as patients who underwent hip surgery, (2) the IV NOA was the “intervention”, (3) the group that did not use IV NOAs was the “comparator”, and (4) “outcomes” were provided for all treatment outcomes. Studies were excluded if the following criteria applied: (1) investigation of data on procedures other than hip surgery or mixed types of surgery that could not be distinguished, (2) non-original articles, (3) articles irrelevant to the research question, and (4) duplicate articles from the same research group. When study populations overlapped, we selected the publication with the largest population for the meta-analysis.

At each stage of article selection, the κ-value was calculated to determine inter-reviewer agreement regarding study selection. Agreement between reviewers was correlated a priori with κ-values as follows: κ = 1 corresponded to “perfect” agreement, 1.0 > κ ≥ 0.8 to “almost perfect” agreement, 0.8 > κ ≥ 0.6 to “substantial” agreement, 0.6 > κ ≥ 0.4 to “moderate” agreement, 0.4 > κ ≥ 0.2 to “fair” agreement, and κ < 0.2 to “slight” agreement. Disagreements at each stage were resolved by discussion between the two investigators to reach a consensus or by discussion with a third investigator when a consensus could not be reached.

### 2.3. Data Extraction

For qualitative data synthesis, the following information and variables were extracted using a standardized form: (1) study design, (2) the country in which the investigation took place, (3) mean patient age, (4) sex, (5) type of hip surgery, (6) number of patients in each IV NOA and control group, (7) types of NOA, (8) pain management protocol between IV NOA group and control group, and (9) outcomes investigated.

For the meta-analyses, the following data were extracted from the included studies for the IV NOA and control groups: (1) opioid use within postoperative 24 h, (2) opioid use during the entire hospitalization period, (3) mean postoperative VAS score, (4) length of hospital stay, and (5) opioid-related adverse events (nausea and vomiting).

### 2.4. Risk-of-Bias Assessment Tool

We assessed the methodological quality of the included studies using the methodological index for non-randomized studies (MINORS) [16], a validated tool for assessing the quality of non-randomized studies. The maximum MINORS checklist score for comparative studies was 24. Two independent reviewers performed the quality assessments. Disagreements were resolved through discussion.

### 2.5. Data Synthesis and Statistical Analyses

For all comparisons, the continuous data were analyzed using standard mean difference (SMD) with 95% confidence intervals (CIs), and for dichotomous data, we calculated odds ratios (ORs) and 95% CIs. We assessed heterogeneity using the I^2^ statistic, considering 25%, 50%, and 75% as low, moderate, and high heterogeneity, respectively. We used forest plots to present the outcomes, pooled estimates of effects, and overall summary effect of each study. We set the statistical significance value at *p* < 0.05. We pooled all data using a random-effects model, as previously described, to avoid overestimation of the study results, particularly in the medical field [17]. We did not perform a test for publication bias because evaluations for publication bias are recommended only when at least 10 studies are included in a meta-analysis [18]. All statistical analyses were performed using the Review Manager (RevMan), version 5.3 (The Nordic Cochrane Centre, The Cochrane Collaboration, 2014; Copenhagen, Denmark).

## 3. Results

### 3.1. Article Identification

**Figure 1:**
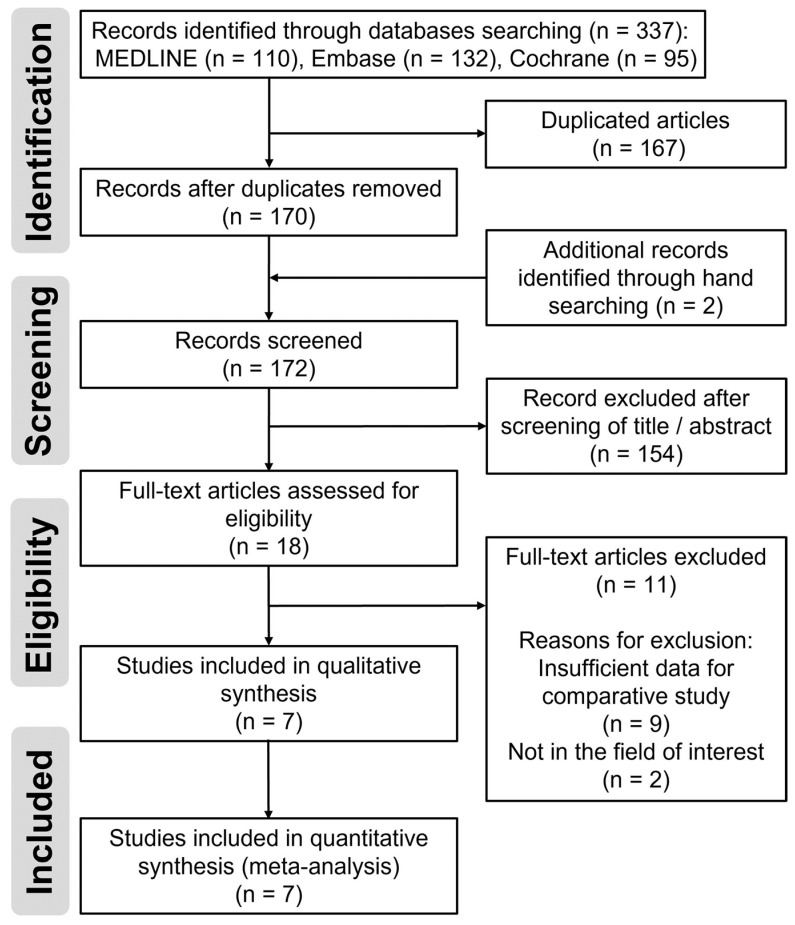
Flow diagram for the identification and selection of studies included in the meta-analysis.

Details of the study identification and selection processes are summarized in Figure 1. The initial electronic literature search yielded 337 articles. After removing 167 duplicates, 170 articles remained. We found two additional articles by manual searching, and 172 articles were screened. Of these, we excluded 154 articles after screening their titles/abstracts, and 11 articles were also excluded step-by-step after full-text review. Finally, seven studies [5,10,19,20,21,22,23] were eligible for qualitative and quantitative data synthesis. The κ-values between the two reviewers were 0.810 at the title review stage and 0.883 at the abstract review stage, indicating near-perfect agreement; this value was 1.000 at the full-text review stage, indicating perfect agreement.

### 3.2. Study Characteristics and Qualitative Synthesis

One study [10] was prospectively designed, two studies [5,19] were retrospective, and the remaining four articles [20,21,22,23] reported a RCT. The majority of studies were conducted in the U.S.; others were conducted in the UK, Japan, and China, respectively. The studies included a total of 953 patients who underwent hip surgery, with 478 patients receiving IV NOA treatment and 475 who did not. Patient mean age ranged from 54.1 years in a study from China to 82.6 years in a study from the U.S. Overall, the studies included more female than male patients, except in two studies [20,21]. Three studies [5,10,19] analyzed patients who underwent hip surgery for hip fractures, and the remaining four studies [20,21,22,23] investigated patients who underwent total hip arthroplasty.

The IV NOA protocol consisted of IV acetaminophen in five studies [5,10,19,21,22] and IV parecoxib in two studies [20,23]. Of the five studies using an IV acetaminophen protocol, one [21] investigated IV acetaminophen as a single dose. IV or PCA opioid was the dominant protocol for the control group in the respective studies, and two [19,22] of these also included oral acetaminophen and oral opioids. Pain intensity was investigated in all studies; in five studies [5,10,19,22,23] a VAS score system was used; of these, one [23] did not provide the VAS score data, although a graph alone; in one study, a five-point pain relief score was used; in one study, pain intensity difference (PID) was scored on a four-point scale or pain relief (PAR) was rated on a five-point scale. Opioid use was investigated as an outcome in all of the studies. The additional details and outcomes investigated in each study are shown in Table 1.

### 3.3. The Result of Risk of Bias Assessment

The mean MINORS score for methodological quality assessment was 19.3/24 (range: 15–22) (Table 1). Regarding the eight main evaluation parameters, all seven studies clearly addressed the aim of their analysis (item 1: a clearly stated aim) and included consecutive patients appropriately (item 2: inclusion of consecutive patients). A two-point deduction was applied to two retrospective studies [5,19] because of their retrospective design (item 3: prospective collection of data), and a two-point deduction was applied to three studies [5,10,19] because of the lack of a prospective calculation of the sample size (item 8: prospective calculation of the study size). All studies addressed the criteria that we used to evaluate the main outcomes of interest for this analysis (item 4: endpoints appropriate to the aim of the study). A two-point deduction was applied to three studies [5,10,19] because the investigators did not conduct unbiased assessments of their study endpoints (item 5: unbiased assessment of the study endpoint). A one-point deduction was applied to all articles because the authors did not describe the length of follow-up. A further point deduction was applied to one study [23] because of the documented lack of follow-up after hospital discharge despite the necessity (item 6: follow-up period appropriate to the aim of the study). A two-point deduction in the follow-up loss parameter was applied to one study [23] because of the apparent follow-up loss after discharge. A one-point deduction was applied to the remaining six studies because the authors did not provide any explanation on the follow-up schedule (item 7: loss to follow-up rate below 5%). A two-point deduction was applied to three studies [5,10,19] because of the lack of prospective calculation of the sample size (item 8: prospective calculation of the study size). A one-point deduction was applied to one study [19] because the control and studied groups were not managed during the same time period (item 10: contemporary groups). No deductions were made from the remaining additional criteria domains (item 9: an adequate control group, item 11: baseline equivalence of groups, item 12: adequate statistical analyses).

### 3.4. Meta-Analysis

#### 3.4.1. Opioid Use

Six studies [5,10,19,20,21,22] evaluated opioid use within 24 h following surgery. Compared with the control group, the IV NOA administration group had a lower opioid use within 24-h following hip surgery (SMD, −0.48; 95% CI, −0.66 to −0.30; *p* < 0.01). Heterogeneity was low to moderate (I^2^ = 34%).

**Figure 2:**
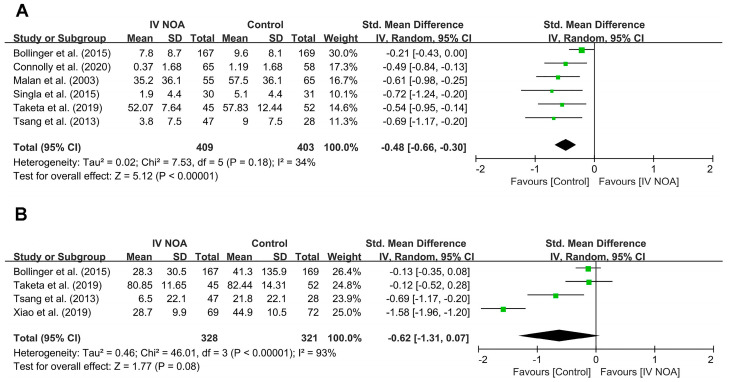
Forest plot showing the opioid use in the IV NOA group and the control group within the first postoperative 24 h (**A**) and during the entire hospitalization period (**B**) following hip surgery.

Four studies [10,19,22,23] evaluated total opioid use during the entire hospitalization period. We failed to reveal a difference in opioid use between the IV NOA group and the control group during the entire hospitalization period with high heterogeneity (SMD, −0.62; 95% CI, −1.31 to 0.07; *p* = 0.08; I^2^ = 93%). A forest plot and further details are shown in Figure 2.

#### 3.4.2. Postoperative VAS Score

**Figure 3:**
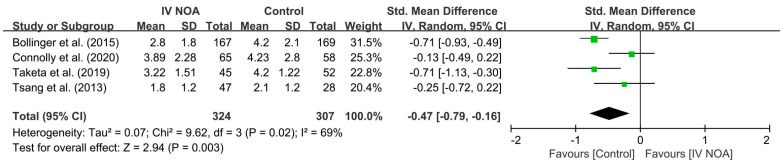
Forest plot showing the postoperative mean VAS score in the IV NOA group and the control group.

Four studies [5,10,19,22] evaluated the mean postoperative VAS score after hip surgery. In a pooled analysis, the control group showed a higher postoperative VAS score than the IV NOA group (SMD, −0.47; 95% CI, −0.79 to −0.16; *p* < 0.01). Heterogeneity was moderate (I^2^ = 69%). A forest plot and further details are shown in Figure 3.

#### 3.4.3. Length of Hospital Stay

**Figure 4:**
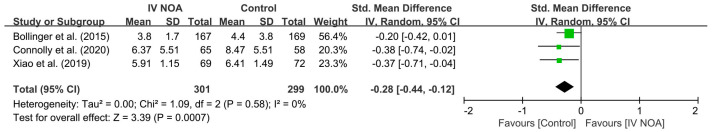
Forest plot showing the length of hospital stay following hip surgery in the IV NOA group and the control group.

Three studies [5,19,23] compared the length of hospital stay between the IV NOA group and the control group. In a pooled analysis, the control group showed longer hospital stays than the IV NOA group with low heterogeneity (SMD, −0.28; 95% CI, −0.44 to −0.12; *p* < 0.01; I^2^ = 0%). A forest plot and further details are shown in Figure 4.

#### 3.4.4. Opioid Related Adverse Event: Nausea and Vomiting

**Figure 5:**
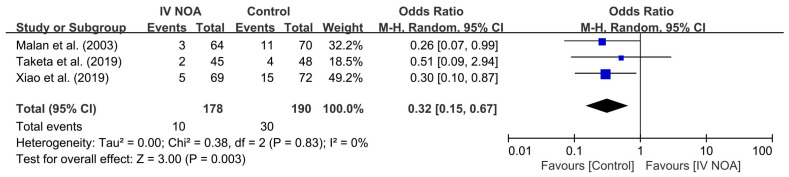
Forest plot showing the occurrence of nausea and vomiting following hip surgery between the IV NOA group and the control group.

Three studies compared the incidence of postoperative nausea and vomiting as an opioid-related adverse event. In a pooled analysis, nausea and vomiting were reported in 5.6% (10/178) of patients in the IV NOA group and 15.8% (30/190) of patients in the control group. The control group showed a higher incidence of nausea and vomiting compared with the control group (OR, 0.32; 95% CI, 0.15 to 0.67; *p* < 0.01). Heterogeneity was low (I^2^ = 0%). A forest plot and further details are shown in Figure 5.

## 4. Discussion

This meta-analysis demonstrated that IV NOA administration may reduce opioid use within 24-h after hip surgery, decreasing the average postoperative VAS pain score, shortening hospitalization, and reducing the occurrence of nausea and vomiting, which are well-recognized opioid adverse events.

As part of efforts to reduce opioid usage for postoperative pain management, there is growing attention to the use of multimodal analgesia strategies. Among these strategies, there have been an increasing number of studies on the effectiveness of IV NOA. Following a previous prospective cohort study comparing 179 surgical procedures [24], orthopedic surgery is recognized as belonging to a group with higher postoperative pain intensity compared to other minor soft tissue surgeries. However, despite this fact, research on IV NOA in the orthopedic field is relatively scarce compared to other areas. In 2022, in the field of orthopedic surgery, Li et al. reported a meta-analysis about the effects of IV parecoxib following overall orthopedic surgery [25]. They finally included 27 articles that dealt with general orthopedic surgery, but only three of them addressed hip arthroplasty. Although the conclusion that morphine consumption within 24 h after surgery was significantly lower in the IV parecoxib treatment group compared to the control group was similar to the findings of our current study, there is a limitation in terms of medication as well, as the study was limited to parecoxib.

In our current study, we confirmed that the use of IV NOA helped reduce opioid consumption within 24 h, but we failed to confirm a decrease in total opioid consumption during the entire hospitalization period. These results suggest that although IV NOA may be effective for short-term pain control within 24 h, it may not have an impact on overall opioid consumption. However, in our opinion, there is a possibility of study bias. This could be due to the limited size of the study population, as only four studies [10,19,22,23] provided data on total opioid consumption. Indeed, compared to opioid consumption within 24 h, total opioid use during the entire hospitalization period falls under high heterogeneity with an I^2^ = 93%, indicating a significant likelihood of bias in the results. Furthermore, there was also considerable variability in the hospitalization period (ranging from 2 to 14 days) among the four studies measuring opioid use during the entire hospitalization period [10,19,22,23].

In the course of this meta-analysis, we identified six papers [5,10,19,22,23,25] comparing postoperative VAS scores between an IV NOA treatment group and a control group. Of these, only four papers had sufficient data for comparative studies, and two papers were not included in our pooled analysis. Hynes et al. [26] conducted a double-blind RCT with 40 patients in the IV propacetamol group, 40 patients in the intramuscular diclofenac group, and 40 patients in the placebo group to investigate postoperative orthopedic pain. In this study, both the IV propacetamol group and the intramuscular diclofenac group showed a significant reduction in VAS scores compared with the placebo group. Similarly, in a double-blind RCT conducted by Xiao et al. [23] involving 69 patients in the IV parecoxib group and 72 patients in the control group, VAS scores decreased more significantly within 48 h in the IV NSAID treatment group during rest and movement. These findings are consistent with the results of our meta-analysis.

Our pooled analysis showed a short length of hospital stay in the IV NOA group compared with the control group. A number of studies have reported an association between post-operative pain and an increased length of hospital stay. Morrison et al. [27] found that postoperative pain was significantly associated with an increased length of hospital stay in patients undergoing surgery for a hip fracture. Elsamadicy et al. demonstrated that the appropriate choice of immediate post-operative pain medication can affect the hospital course for patients following orthopedic spine surgery [28]. The results of the present study are consistent with these findings.

We performed a meta-analysis of the occurrence of nausea and vomiting. Consistent with a previous study [29], we found that up to 40% of patients may experience nausea and 15–25% of patients may experience vomiting after opioid administration. The use of IV NOA can reduce opioid-related adverse events such as nausea and vomiting by reducing acute opioid use within 24 h after surgery.

Our study has several limitations. First, there was a lack of standardization in measuring opioid use >24 h. While most of the included papers measured opioid use precisely at 24 h after surgery, others measured acute use between 4 and 6 h postoperatively [21]. This variability in the measurement of the opioid use window is a potential source of bias. Second, inconsistent opioid administration methods were used. In some patients, IV opioids were administered, while others were treated with oral opioid formulations. This is an unavoidable aspect of the retrospective nature of meta-analyses. Nevertheless, a meta-analysis is an appropriate method to generate a high level of evidence in rare conditions, suggesting that our synthetic results are meaningful. Finally, this meta-analysis did not include studies using the most recently developed medications, such as the combination of IV acetaminophen and IV NSAIDs. Future large-scale prospective studies are required to draw definitive conclusions.

## 5. Conclusions

This meta-analysis demonstrated more favorable postoperative outcomes in patients treated with IV NOA after hip surgery than those in controls in terms of postoperative 24-h opioid use, pain score, hospitalization period, and occurrence of opioid-related adverse events.

## Figures and Tables

**Table 1:** Study design, demographic data, study details of included studies.

Author (Year)	Study Design	Country	Patient Mean Age (yrs)	Sex (%)		Hip Surgery Type	No. of Sample Size		NOA Type	Pain Management Protocol		Outcome Investigated	MINORS Score
				Male	Female		IV NOA	Control		IV NOA	Control		
Bollinger et al. (2015) [19]	RCS	U.S.	82.6	27.1	72.9	Hip Op.	167	169	IV AAP	IV AAP multiple (1 g, 3 times) Oral AAP Oral narcotics IV morphine	Oral AAP Oral narcotics IV morphine	LOS, VAS pain score, Opioid use, Rate of missed PT, Adverse effects, GI disturbance, DD	15
Connolly et al. (2020) [5]	RCS	U.S.	80.1	40.7	59.3	Hip Op.	65	58	IV AAP	IV AAP multiple (1 g, 3 times) Oral oxycodone IV morphine	Oral AAP Oral oxycodone IV morphine	Delirium, Opioid use, VAS pain score, Readmission, Required one to one supervision, LOS, DD	16
Malan et al. (2003) [20]	RCT	U.S.	65.9	53.7	46.3	THA	131 (67 + 64)	70	IV Parecoxib	IV Parecoxib 20 mg or 40 mg PCA morphine	Placebo PCA morphine	Opioid use, pain relief (5-point scale), study med Global evaluation (4-point scale), Adverse effects	22
Singla et al. (2015) [21]	RCT	U.S.	64.1 (study1) 61.0 (study2)	43.5 (study1) 65.6 (study2)	56.5 (study1) 34.4 (study2)	THA	35 (study1) 30 (study2)	34 (study1) 31 (study2)	IV AAP	IV AAP single shot PCA morphine (study1) IV AAP multiple PCA morphine (study2)	Placebo (Saline) PCA morphine	Time to MCID, PID, PAR scores, Rescue medication use, Time to first Opioid use, Opioid use, Safety (TEAEs)	22
Taketa et al. (2019) [22]	RCT	Japan	64.4	17.5	82.5	THA	45	52	IV AAP	IV AAP multiple PCA fentanyl Oral AAP, FNB	PCA fentanyl Oral AAP, FNB	VAS pain score, Opioid use, Adverse effects, AST value, ALT value	22
Tsang et al. (2013) [10]	PCS	UK	80.4	21.3	78.7	Hip Op.	47	28	IV AAP	IV AAP multiple (1 g, 4 times) IV morphine	Oral AAP IV morphine	Opioid use, VAS pain score	18
Xiao et al. (2019) [23]	RCT	China	54.1	41.0	59.0	THA	69	72	IV Parecoxib	IV Parecoxib 40 mg, 4 times PCA morphine	Placebo (Saline) PCA morphine	Opioid use, LOS, Adverse effects, Functional recovery, Bleeding risk, Inflammatory response	20

No., number; RCS, retrospective cohort study; RCT, randomized controlled trial; Op., operation; AAP, acetaminophen; LOS, length of hospital stay; PT, physical therapy; GI, gastrointestinal; DD, discharge disposition; THA, total hip arthroplasty; PCA, pain-controlled analgesia; PAR, pain relief; PID, pain intensity difference; MCID, minimal clinically important difference; FNB: femoral nerve block; TEAEs, treatment-emergent adverse events; NOA, non-opioid analgesics; IV, intravenous.

## Data Availability

The datasets generated and analyzed during the current study are not publicly available due to the presence of personally identifiable patient information. However, they can be obtained from the corresponding author upon reasonable request.

## Appendix A. The Literature Search Algorithm and the Results from Relevant Clinical Studies

**PubMed** (**7 June 2023**)

	**Search Queries**	**Articles#**
#1	hip [Title/Abstract]	169,158
#2	intravenous [Title/Abstract]	321,291
#3	intraoperative [Title/Abstract]	165,212
#4	#2 OR #3	481,086
#5	acetaminophen [Title/Abstract]	18,301
#6	NSAIDs [Title/Abstract]	22,908
#7	Anti-Inflammatory Agents, Non-Steroidal [MeSH Terms]	88,368
#8	#5 OR #6 OR #7	113,005
#9	#4 AND #8	4485
#10	#1 AND #9	110

**Embase** (**7 June 2023**)

	**Search Queries**	**Articles #**
#1	hip:ti,ab,kw	223,922
#2	intravenous:ti,ab,kw	447,004
#3	intraoperative:ti,ab,kw	237,477
#4	#2 OR #3	676,143
#5	acetaminophen:ti,ab,kw	27,459
#6	NSAIDs:ti,ab,kw	41,470
#7	#5 OR #6	66,804
#8	#4 AND #7	4000
#9	#1 AND #8	132

**Cochrane Library (7 June 2023)**

	**Search Queries**	**Articles #**
#1	hip:ti,ab,kw	28,525
#2	intravenous:ti,ab,kw	99,417
#3	intraoperative:ti,ab,kw	32,422
#4	#2 OR #3	125,871
#5	acetaminophen:ti,ab,kw	7156
#6	NSAIDs:ti,ab,kw	5543
#7	#5 OR #6	12,124
#8	#4 AND #7	2168
#9	#1 AND #8	95
